# The effect of antibiotic premedication on postoperative complications following dental extractions

**DOI:** 10.1111/jphd.12634

**Published:** 2024-08-12

**Authors:** Jessina C. McGregor, Geneva M. Wilson, Gretchen Gibson, M. Marianne Jurasic, Charlesnika T. Evans, Katie J. Suda

**Affiliations:** ^1^ Department of Pharmacy Practice, College of Pharmacy Oregon State University Portland Oregon USA; ^2^ Center of Innovation for Complex Chronic Healthcare (CINCCH) Hines Jr. Veterans Affairs Hospital Hines Illinois USA; ^3^ Department of Preventive Medicine, Center for Health Services and Outcomes Research Northwestern University Feinberg School of Medicine Chicago Illinois USA; ^4^ Veterans Health Administration Office of Dentistry Washington DC USA; ^5^ Kansas City University College of Dental Medicine Joplin Missouri United States; ^6^ Department of Health Policy and Health Services Research Boston University Henry M. Goldman School of Dental Medicine Boston Massachusetts USA; ^7^ Center for Health Equity Research and Promotion VA Pittsburgh Health Care System Pittsburgh Pennsylvania USA; ^8^ Department of Medicine University of Pittsburgh School of Medicine Pittsburgh Pennsylvania USA

**Keywords:** antibiotic, antibiotic prophylaxis, dentist, medication safety, prescribing patterns, tooth extraction

## Abstract

**Objectives:**

We aimed to evaluate the association between antibiotic prophylaxis and adverse outcomes following tooth extraction within the Veterans Affairs Healthcare System.

**Methods:**

We conducted a retrospective cohort study of patients undergoing dental extractions in 2015–2019. The primary exposure was antibiotic prophylaxis. The primary outcome was post‐extraction complication within 7 days (e.g., alveolar osteitis and surgical site infection); the secondary outcome was subsequent medical care relating to a post‐extraction oral complication within 7 days. Multivariable logistic regression models assessed the independent effect of antibiotic prophylaxis on each outcome.

**Results:**

Of 385,880 visits with a dental extraction, 122,810 (31.8%) received antibiotic prophylaxis. Overall, 3387 (0.9%) experienced a post‐extraction complication and 350 (0.09%) received medical care relating to a post‐extraction oral complication within 7 days. In multivariable regression, diabetes was a statistically significant (*p* = 0.01) effect modifier of the association between antibiotic prophylaxis and post‐extraction complication. Among visits for patients without diabetes, antibiotic prophylaxis was significantly associated with an increased odds of post‐extraction complication (odds ratio [OR] = 1.25, 95% confidence interval [CI]: 1.13–1.38), but among visits for patients with diabetes no significant effect was observed (OR = 1.03, 95% CI: 0.92–1.15). Antibiotic prophylaxis was not significantly associated with post‐extraction medical care (OR = 1.04; 95% CI: 0.83–1.30).

**Conclusions:**

In this large retrospective cohort, we observed no significant protective effect of antibiotic prophylaxis on post‐extraction complications or subsequent medical care utilization in a setting with low complication rates. These data suggest that use of antibiotic prophylaxis in similar settings may need to be re‐evaluated to minimize unnecessary antibiotic use.

## INTRODUCTION

Dentists provide tooth extractions primarily due to the effects of dental caries and periodontal disease, both of which are infectious diseases that can lead to break down of the tooth, supporting structures, and other local and systemic complications. While the number of remaining teeth throughout the lifespan has risen over time, risk factors such as race, lower education, lower income, and lifestyle choices such as smoking, have lead to subpopulations with comparatively higher rates of tooth loss [1].

Tooth removal in a dental clinical setting is accomplished primarily in an outpatient setting under locally administered anesthesia. In general, the longer and more tissue invasive a procedure, the higher the possible risk of postoperative infection [2]. Post‐extraction complications which are most often documented and studied include pain, swelling, alveolar osteitis (dry socket), and surgical site infection [3, 4, 5].

Systemic antibiotics are provided by dental practitioners with the purpose of preventing post‐extraction complications. A recent analysis in the Veterans Health Administration (VHA) showed only 15% of antibiotic prescriptions were appropriate per guidelines using a narrow definition of appropriate premedication being only patients who required premedication due to cardiac conditions at the highest risk for adverse outcomes [6]. When this definition was expanded to include immunocompromising conditions, tooth extractions, and dental implant placement, the number of appropriate prescriptions based on evidence rose to 72% [6, 7]. This clearly indicates that dental providers are utilizing antibiotics as a preventive measure for postoperative complications, but the data around this practice is mixed or nonexistent for medically compromised patients. In a recently updated Cochrane systematic review utilizing only data relating to the extraction of third molars, the authors noted low‐certainty evidence that antibiotics may reduce the risk of postoperative infection by 66% and dry socket by 34%, however, the effect was uncertain regarding pain and fever [8]. Extraction of third molars is routinely considered surgical, performed in a younger, generally healthy population, and more invasive than other single tooth extractions. None of the included studies addressed this topic in immunocompromised patients. Several past and recent studies have evaluated the effect of antibiotic premedication on post‐extraction complications in healthy adults undergoing routine or nonsurgical extractions. None identified a benefit in providing systemic antibiotics to prevent post‐extraction complications when compared to no antibiotics or a placebo [4, 5, 9, 10].

Veterans Affairs (VA) Dental patients represent an older population, which carry a higher oral and systemic disease burden than the general population. Data show they have a higher rate of caries as well as mental and physical comorbidities such as post‐traumatic stress disorder, depression, and diabetes mellitus [11]. It is feasible that some or many of these disease processes may affect patients postoperative healing process. For these reasons, the aim of this study is to evaluate the effectiveness of antibiotic prophylaxis to prevent localized and systemic post extraction complications in a national population of VA dental patients undergoing extraction procedures.

## METHODS

### Study setting and sample

A retrospective cohort study was performed among all VHA dental visits in which an extraction was performed between January 1, 2015 and December 31, 2019 using data from the VHA Corporate Data Warehouse (CDW), a national, real time relational data repository. Dental extractions were identified by Code on Dental Procedures and Nomenclature (CDT) codes D7140, D7210, D7250, and D7711. Patients who had an implant placed at index visit or within 90 days following the index visit, and those with a dental visit between the mailed antibiotic prescription date and extraction were excluded. Additionally, patients undergoing removal of an impacted tooth (CDT D7220, D7230, D7240, and D7241) at the index visit were excluded. The Edward Hines, Jr. VA Investigational Review Board granted this study expedited approval and was exempt from informed consent.

### Primary exposure variable

The primary exposure of interest was antibiotic prophylaxis. We identified an antibiotic as prophylaxis based on the proximity in time of the dispensed antibiotic to the extraction procedure. In the VHA, medications can be dispensed at an outpatient pharmacy or via mail order from a centralized pharmacy. Thus we identified antibiotic prophylaxis as an antibiotic prescribed and mailed within 30 days prior to the date of the extraction, or an antibiotic dispensed from an outpatient pharmacy within 7 days prior to or on the date of the extraction. In the event that multiple eligible antibiotic prescriptions were identified, the antibiotic dispense date closest to the extraction date was selected as the antibiotic prophylactic agent.

### Outcome variables

The primary outcome of interest was post‐extraction complication, which was defined as the occurrence of oral infection, dry socket, or fever within 7 days following extraction. ICD‐10‐CM codes were used to identify oral infections (K046, M272, M2749, M273, K048, K122, and M8708), dry socket at the extraction site (M27.3), or fever (R50.9). ICD‐9‐CM codes prior to October 2015 were converted to ICD‐10‐CM. The secondary outcome was receipt of subsequent medical care within 7 days post extraction due to oral infection/complication related reasons.

### Covariates

Data were collected pertaining to patient demographics, comorbidities, medical history, visit characteristics, and extraction characteristics for considerations as potential confounders [12]. Comorbidities were identified using ICD‐10‐CM codes according to Selim et al. from up to 1 year prior to the extraction [13]. Cardiac conditions were defined upon risk for infective endocarditis to align with dental antibiotic prophylaxis guidelines [14, 15]. Patients were classified as having a history of oral infection if identified by diagnosis codes between −31 and −365 days relative to the date of extraction. Patients with oral infection diagnosis codes identified from −30 days to the date of extraction were classified as having oral infection at baseline. Immunosuppression was measured by the use of systemic immunocompromising medications (e.g., chemotherapy) or by the presence of immunocompromising conditions within a year pre‐extraction [6]. The full list of immunocompromising conditions/medications can be found in Table S1. Poorly controlled diabetes was identified based on an average HbA1C from the past year of 8% or higher.

### Statistical analysis

Summary measures were used to describe the cohort characteristics, including the frequency of exposure and outcome variables. The association between the antibiotic prophylaxis and each outcome variable was modeled using logistic regression analysis. Variables with a *p* < 0.20 in univariable analysis were considered for inclusion in the final multivariable model. The final parsimonious model contained all variables with *p* < 0.05 and any confounders, which were identified as resulting in a 20% or greater change in the odds ratio (OR) for antibiotic prophylaxis. Additionally, an interaction term with diabetes was tested for inclusion in the model.

We performed a sensitivity analysis in which visits with an antibiotic prescribed by a medical provider were excluded. The final parsimonious regression models for each outcome in the primary analysis were also fitted to the restricted cohort for the sensitivity analysis.

## RESULTS

We identified 385,880 visits among 269,003 unique patients for inclusion in the study cohort. Table 1 presents summary statistics of all included variables; data are stratified by exposure status (antibiotic prophylaxis) since multiple outcomes were evaluated. A similar number of extraction visits were identified across all years of the study ranging from 21.1% of visits occurring in 2015 to 18.2% occurring in 2019. The majority of visits occurred within the Southern region of the U.S. (45.5%). Most visits were for male patients (92.9%) aged 65–79 years (47.7%) and 45–64 years (31.9%). While 66.7% of visits were for patients of white race, 26.3% were for patients of black race. Half of the visits (50%) were for patients with diabetes.

**TABLE 1:** Characteristics of dental extraction cohort visits (*n* = 385,880 visits among 269,003 unique patients).

	Antibiotic prophylaxis, *n* = 122,810	No antibiotic prophylaxis, *n* = 263,070	Total cohort, *n* = 385,880	*p*‐value
Post‐extraction complication within 7 days	1272 (1.0%)	2115 (0.8%)	3387 (0.9%)	<0.0001
Fever	126 (0.1%)	228 (0.1%)	353 (0.1%)	
Oral infection	1151 (0.9%)	1895 (0.7%)	3046 (0.8%)	
Dry socket	436 (0.4%)	1215 (0.5%)	1651 (0.4%)	
Medical care within 7 days	118 (33.7%)	232 (0.09%)	350 (0.09%)	0.448
Year				
2015	25,806 (21%)	55,440 (21.1%)	81,246 (21.1%)	
2016	26,454 (21.5%)	54,050 (20.5%)	80,504 (20.9%)	<0.0001
2017	25,544 (20.8%)	52,706 (20%)	78,250 (20.3%)	0.0002
2018	23,556 (19.2%)	52,203 (19.8%)	75,759 (19.6%)	0.0043
2019	21,450 (17.5%)	48,671 (18.5%)	70,121 (18.2%)	<0.0001
US Census Bureau Region				
Northeast	18,541 (15.1%)	41,461 (15.8%)	60,002 (15.5%)	
Midwest	26,444 (21.5%)	43,807 (16.7%)	70,251 (18.2%)	<0.0001
South	53,093 (43.2%)	122,433 (46.5%)	175,526 (45.5%)	0.0027
West	24,732 (20.1%)	55,369 (21%)	80,101 (20.8%)	0.9214
Age (years)				
18–44	18,951 (15.4%)	35,315 (13.4%)	54,266 (14.1%)	<0.001
45–64	40,644 (33.1%)	82,495 (31.4%)	123,139 (31.9%)	<0.001
65–79	56,673 (46.1%)	127,348 (48.4%)	184,021 (47.7%)	<0.001
≥80	6542 (5.3%)	17,912 (6.8%)	24,454 (6.3%)	
Male gender	113,813 (92.7%)	244,495 (92.9%)	358,308 (92.9%)	0.0029
Race^a^				
White	81,183 (66.1%)	176,210 (67%)	257,393 (66.7%)	
Black	32,776 (26.7%)	68,624 (26.1%)	101,400 (26.3%)	<0.001
Native American/Alaskan	1019 (0.8%)	2293 (0.9%)	3312 (0.9%)	0.3411
Native Hawaiian/Pacific Islander	1221 (1%)	2503 (1%)	3724 (1%)	0.1041
Asian	1173 (1%)	2329 (0.9%)	3502 (0.9%)	0.0134
Multiracial	1361 (1.1%)	2775 (1.1%)	4136 (1.1%)	0.0608
Missing	4077 (3.3%)	8336 (3.2%)	12,413 (3.2%)	0.0023
Ethnicity				
Non‐Latine	110,637 (90.1%)	240,725 (91.5%)	351,362 (91.1%)	
Latine	9882 (8%)	17,340 (6.6%)	27,222 (7.1%)	<0.001
Missing	2291 (1.9%)	5005 (1.9%)	7296 (1.9%)	0.8738
Diabetes	63,423 (51.6%)	129,485 (49.2%)	192,908 (50%)	<0.0001
Poorly controlled diabetes within the past year	14,578 (11.9%)	25,683 (9.8%)	40,261 (10.4%)	<0.0001
Immunocompromised	9861 (8.1%)	17,163 (6.5%)	27,024 (7%)	<0.001
Cardiac condition	20,590 (16.8%)	40,296 (15.3%)	60,886 (15.8%)	<0.0001
Smoking history (tobacco)				
Never smoked	20,502 (16.7%)	42,013 (16%)	62,515 (16.2%)	
Current smoker	42,226 (34.4%)	92,993 (35.3%)	135,219 (35%)	<0.0001
Past smoker	20,154 (16.4%)	42,696 (16.2%)	62,850 (16.3%)	0.0059
Missing	39,928 (32.5%)	85,368 (32.5%)	125,296 (32.5%)	<0.0001
Hepatitis	1396 (1.1%)	2942 (1.1%)	4338 (1.1%)	0.6139
Cancer	6897 (5.6%)	13,954 (5.3%)	20,851 (5.4%)	<0.0001
Skin cancer	798 (0.6%)	1661 (0.6%)	2459 (0.6%)	0.5036
Thyroid disorder	3510 (2.9%)	7559 (2.9%)	11,069 (2.9%)	0.7908
Gout	2016 (1.6%)	4171 (1.6%)	6187 (1.6%)	0.1966
Anemia	4602 (3.7%)	8981 (3.4%)	13,583 (3.5%)	<0.0001
Seizure	639 (0.5%)	1356 (0.5%)	1995 (0.5%)	0.8444
Cataract	9312 (7.6%)	20,306 (7.7%)	29,618 (7.7%)	0.1381
Hypertension	23,794 (19.4%)	49,315 (18.7%)	73,109 (18.9%)	<0.0001
Myocardial infarction	1122 (0.9%)	2154 (0.8%)	3276 (0.8%)	0.0028
Angina	645 (0.5%)	1252 (0.5%)	1897 (0.5%)	0.0415
Irregular heartbeat	4584 (3.7%)	9347 (3.6%)	13,931 (3.6%)	0.0054
Congestive heart failure	2808 (2.3%)	5559 (2.1%)	8367 (2.2%)	0.0006
Stroke	1725 (1.4%)	3740 (1.4%)	5465 (1.4%)	0.676
Transient ischemic attack	428 (0.3%)	904 (0.3%)	1332 (0.3%)	0.8101
Pulmonary vascular disease	2202 (1.8%)	4576 (1.7%)	6778 (1.8%)	0.2382
Chronic obstructive pulmonary disease	6801 (5.5%)	14,594 (5.5%)	21,395 (5.5%)	0.9109
Peptic ulcer	457 (0.4%)	916 (0.3%)	1373 (0.4%)	0.245
Bowel disease	320 (0.3%)	715 (0.3%)	1035 (0.3%)	0.53
Infectious disease	1497 (1.2%)	3263 (1.2%)	4760 (1.2%)	0.5748
Gall bladder disorder	627 (0.5%)	1251 (0.5%)	1878 (0.5%)	0.1455
Urinary tract infection history	1624 (1.3%)	3315 (1.3%)	4939 (1.3%)	0.1091
Benign prostatic hyperplasia	4942 (4%)	10,684 (4.1%)	15,626 (4%)	0.5383
Prostatitis	229 (0.2%)	510 (0.2%)	739 (0.2%)	0.6244
Arthritis, nonrheumatoid and nonosteo	2462 (2%)	4880 (1.9%)	7342 (1.9%)	0.0015
Rheumatoid arthritis	592 (0.5%)	1177 (0.4%)	1769 (0.5%)	0.1379
Osteoarthritis	9540 (7.8%)	19,847 (7.5%)	29,387 (7.6%)	0.0147
Low back pain	14,370 (11.7%)	30,346 (11.5%)	44,716 (11.6%)	0.1342
Hip fracture	2093 (1.7%)	4393 (1.7%)	6486 (1.7%)	0.4393
Schizophrenia	2039 (1.7%)	4978 (1.9%)	7017 (1.8%)	<0.0001
Bipolar disease	3451 (2.8%)	7264 (2.8%)	10,715 (2.8%)	0.3902
Depression	7160 (5.8%)	14,488 (5.5%)	21,648 (5.6%)	<0.0001
Anxiety	6543 (5.3%)	13,573 (5.2%)	20,116 (5.2%)	0.0285
Alcohol use disorder	5288 (4.3%)	11,401 (4.3%)	16,689 (4.3%)	0.6905
Post‐traumatic stress disorder	13,440 (10.9%)	29,980 (11.4%)	43,420 (11.3%)	<0.0001

^a^ Race and ethnicity was self‐identified. Veterans with multiple races selected were categorized as multiracial.

The majority of patients had only one tooth extracted (61.1%) and 59.4% had only nonsurgical extractions performed at their index visit. Dental providers performed 91.8% of all extractions in this cohort, with the remaining extractions being performed by oral surgeons (8.0%) and residents (0.2%). There was a low prevalence of oral infection history (2.9%), implant history (1%), and oral infection at baseline (1.2%). Table 2 presents extraction characteristics stratified by receipt of antibiotic prophylaxis. Antibiotic prophylaxis was received more frequently by patients that underwent surgical extraction compared to nonsurgical extractions (37.9% vs. 27.7%, respectively).

**TABLE 2:** Frequency of antibiotic prophylaxis by dental extraction procedure characteristics.

	Antibiotic prophylaxis, *n* = 122,810
Extraction type	
No surgical extraction	63,473 (27.7%)
Any surgical extraction	59,337 (37.9%)
Number of tooth extractions	
1	91,620 (38.8%)
2	22,872 (24.9%)
3+	8318 (14.3%)
Extraction provider type*	
Dentist	111,873 (91.1%)
Resident	179 (0.1%)
Oral surgeon	10,758 (8.8%)
History of dental implant	1670 (42.6%)
History of oral infections	3818 (33.9%)
Oral infection at baseline	2327 (48.8%)

* Note that the table presents the frequency of visits in which antibiotic prophylaxis was prescribed by the extraction provider type. The extraction provider type may differ from the prescribing provider.

There were 122,810 (31.8%) visits in which patients received antibiotic prophylaxis. Table 3 presents the characteristics of the prophylactic regimens received by these patients. Amoxicillin was the most frequent agent prescribed (69%) followed by clindamycin (16%).

**TABLE 3:** Characteristics of antibiotic prophylaxis received (*n* = 122,801).

	*n* (%)
Type of antibiotic prophylaxis	
Amoxicillin	84,767 (69.0%)
Clindamycin	19,684 (16.0%)
Amoxicillin clavulanate	9663 (7.9%)
Penicillin	9348 (7.6%)
Doxycycline	2299 (1.9%)
Azithromycin	2251 (1.8%)
Cephalosporin	1715 (1.4%)
Other antibiotics	366 (0.3%)
Prescriber type	
Dental provider only	106,885 (87.0%)
Any medical provider	15,925 (13.0%)

Overall, 3387 (0.9%) experienced a post‐extraction complication within 7 days. Of these, 1272 (1%) complications occurred in patients who had received antibiotic prophylaxis and 2115 (0.8%) in patients who did not receive prophylaxis. In multivariable regression analysis (Table 4), diabetes was identified as a statistically significant (*p* = 0.01) effect modifier of the association between antibiotic prophylaxis and post‐extraction complication, thus an interaction term was included in the final multivariable regression model. Among visits for patients without diabetes, antibiotic prophylaxis was significantly associated with an increased odds of post‐extraction complication (OR = 1.25, 95% confidence interval [CI]: 1.13–1.38), but among visits for patients with diabetes no significant effect was observed (OR = 1.03, 95% CI: 0.92–1.15; Table 4). In a sensitivity analysis excluding patients who had received antibiotic prescriptions from medical providers, a similar pattern was observed (Table 4).

**TABLE 4:** Independent association between antibiotic prophylaxis and post‐extraction complication within 7 days in the full cohort and sensitivity analysis.^a^

	Post‐extraction complication within 7 days	
	Odds ratio	95% confidence interval
Primary analysis^b^		
Antibiotic prophylaxis among patients without diabetes	1.25	1.13–1.38
Antibiotic prophylaxis among patients with diabetes	1.03	0.92–1.15
Sensitivity analysis^c^		
Antibiotic prophylaxis among patients without diabetes	1.14	1.02–1.27
Antibiotic prophylaxis among patients with diabetes	0.97	0.86–1.08

^a^ Effect estimates are stratified by diabetes status as diabetes was identified as a significant effect modifier.

^b^ Analysis is adjusted for age, gender, race, ethnicity, region, immunosuppressant use, smoking history, cataract, angina, stroke, pulmonary vascular disease, nonrheumatoid/‐osteo arthritis, hip fracture, anxiety, oral infection history, oral infection at baseline, extraction type, extraction number.

^c^ Sensitivity analysis includes only visits in which patients received an antibiotic prescription from a medical provider within the exposure window (*n* = 106,885).

In the 7 days post‐extraction, only 350 (0.09%) patients received medical care relating to a post‐extraction oral complication. In multivariable regression analysis, antibiotic prophylaxis was not significantly associated with post‐extraction medical care (OR = 1.04; 95% CI: 0.83–1.30; Table 5). While effect modification with diabetes was assessed, no significant interaction was identified. In the sensitivity analysis excluding patients with antibiotics prescribed by medical (i.e., nondental) providers, antibiotic prophylaxis remained not associated with post‐extraction medical care (OR = 0.92; 95% CI: 0.72–1.18; Table 5).

**TABLE 5:** Independent association between antibiotic prophylaxis and post‐extraction medical care for an oral infection related reasons within 7 days in the full cohort and sensitivity analysis.

	Post‐extraction medical care for oral infection related reasons	
	Odds ratio	95% confidence interval
Full cohort^a^		
Antibiotic prophylaxis	1.04	0.83–1.30
Sensitivity analysis^b^		
Antibiotic prophylaxis	0.92	0.72–1.18

^a^ Analysis is adjusted for year, age, smoking history, extraction provider.

^b^ Sensitivity analysis excludes visits in which patients received an antibiotic prescription by a medical provider within the exposure window (*n* = 106,885).

## DISCUSSION

In this cohort of Veteran dental patients, the identified 7‐day postoperative complication incidence was below 1% over the 5‐year period. This included both surgical and nonsurgical extractions, indicating varying levels of procedure difficulty and invasiveness, but third molar extractions were excluded. The incidence of complications observed in our study was in the lower range of what has been reported among the much smaller randomized control trials in healthy adults, where the incidence of infectious complications ranged from 0% to 14% [4, 5, 9, 10]. The variability in complication rates across published studies may be indicative of differences in measurement, protocol, procedure types included, or patient population. Chart review of a sample of patients who did not receive antibiotics suggested some misclassification in identification of antibiotic use, but very few patients with the complication outcome variable were missed [16].

The use of antibiotics either pre or peri‐procedure showed no positive effect on the outcome of reduced postoperative infection or dry socket, compared to those who did not receive an antibiotic prescription. This finding is consistent with previous studies in healthy adults undergoing nonsurgical extractions, where all found no benefit to the use of antibiotics to prevent postoperative infection [4, 5, 9, 10].

It has been established that Veterans who receive comprehensive care through the VA system carry a higher dental caries and systemic disease burden [11]. Most previous studies evaluated antibiotic prophylaxis for third molar or simple extractions in healthy adults. As remarked by Lodi et al., the question regarding the need for antibiotic prophylaxis for extractions in medically compromised patients is still unanswered [8]. Our results, comprised of the largest extraction study to date, can inform this gap in the evidence. First, for patients identified as immunocompromised we observed an increased odds of complication within 7 days of extraction in unadjusted analysis. However, an immunocompromised state was not included in the final multivariable model as it was not a confounder of the association between antibiotic prophylaxis and post‐extraction complication and was also not a significant predictor of complications. The rate of diabetes diagnosis within this population was at 50%, which is significantly higher than what is diagnosed in the general population (38% of the adult population) [17]. This is consistent with prior work which demonstrated higher rates of comorbidities in the Veteran population compared to the general population, indicating a population of higher medical complexity [11, 18]. Diabetes was found to be an effect modifier for the association between use of antibiotic prophylaxis and post‐extraction complication. However, as seen in the final model, providing antibiotic prophylaxis did not prevent post‐extraction complications. Our results did identify a negative effect—diabetic patients that received antibiotic prophylaxis had a significantly increased odds for post‐extraction complications. One explanation for this unexpected finding is that providers identified these patients as high‐risk for complications and antibiotic prophylaxis did not mitigate this risk. Regardless, our results demonstrate that the use of antibiotic prophylaxis, even for patients that are more medically compromised, does not appear to be associated with a lower post‐extraction complication rate.

As noted in Table 2, VA dentists provided antibiotics most often when a higher risk for postoperative complications was expected, such as inclusion of a surgical extraction and notation of an oral infection at baseline. The type of antibiotic utilized mirrors other studies, with 69% of the antibiotics being for amoxicillin. Clindamycin was the second most utilized at 16%, which has since been discouraged from use due to the higher propensity of adverse events such as *Clostridioides difficle* infection [19].

This study has several limitations. First, the study sample is primarily male, therefore this data may not be generalizable to females. Second, our study identified post‐extraction complications using medical and dental records. Therefore only clinically relevant complications were identified. Furthermore, using only ICD9/10 diagnoses could over‐estimate the outcome variable of localized oral infection, as noted by a chart review of a sample of Veterans that received a tooth extraction and no antibiotic prophylaxis [16]. This primarily occurred due to carrying over a code to subsequent visits where additional procedures or monitoring occurred for localized oral infection. However, the same chart review noted few patients with complications were excluded using our algorithm to identify post‐extraction complications. Finally, there may be other risk factors for postoperative complications that were not accounted for in this model.

Our results combined with prospective randomized controlled clinical trials should inform the discontinuation of antibiotic prophylaxis to prevent post‐extraction complications, even in patients with diabetes or other medically complex patients. Of all antibiotics prescribed by dentists, one in five are prescribed to prevent postsurgical complications [20]. The majority of antibiotic use in the US is in outpatients in the community, and dentists are high prescribers of antibiotics [21, 22]. Thus, eliminating antibiotic prophylaxis for an unnecessary indication could significantly reduce community antibiotic use [21, 23]. Professional organizations should, for the first time, publish clinical practice guidelines to provide evidence‐based recommendations. This is critical as antibiotic prophylaxis prescribed by dentists have been associated with *C*. *difficile*, allergic reactions, and increased health care utilization and any antibiotic use is associated with antimicrobial resistance [24, 25]. Despite prior guideline changes, little change in antibiotic prescribing has occurred [26]. Dentists and dental clinics are included in recommendations issued by the Centers for Disease Control and Prevention (CDC) to improve antibiotic use [27, 28]. While isolated to specific clinics or providers, antibiotic stewardship efforts to improve prescribing by dentists have shown to successfully improve antibiotic use [29, 30, 31]. Future research should study the de‐implementation of antibiotic prophylaxis to prevent post‐extraction complications.

## CONCLUSION

In this large study with a high percentage of older and medically compromised patients who underwent tooth extraction surgery at VA medical centers, the postoperative complication rate was less than 1%. Utilization of pre‐ or periprocedural antibiotics to reduce localized or systemic postoperative complications from tooth extraction procedures did not provide any benefit. Therefore, the benefit of antibiotic prophylaxis is unlikely to exceed the risk of antibiotic‐associated adverse events. Dental providers are encouraged to review their protocol for the use of antibiotic prophylaxis for these procedures and reserve use for only those patients who have a current systemic infection for which antibiotic treatment is indicated.

## Supporting information


**Appendix S1.** Supporting information.
