# Supplementary material for: The effect of antibiotic premedication on postoperative complications following dental extractions
Source: J Public Health Dent. 2024 Aug 12;84(4):343–50. doi: 10.1111/jphd.12634 (PMC11619531; doi:10.1111/jphd.12634)
Supplement: Supplementary file 1 — Appendix S1. Supporting information. [file JPHD-84-343-s001.docx]

| Appendix 1. Definitions of Highly Immunocompromising Conditions or Drugs | |
| --- | --- |
| Conditions | |
| Patient has any: | Congenital agranulocytosis |
|  | Blood stem cells transfusion |
|  | Bone marrow status, transplant, or transfusion |
|  | Bone marrow transplant rejection or failure |
|  | Complications of transplant |
|  | Cord blood stem cell transplant or transfusion |
|  | Graft versus host disease |
|  | Hematopoietic stem cell transplant or transfusion |
|  | Hypogammaglobulinemia |
|  | Neutropenia |
|  | Rheumatoid arthritis |
|  | Selective immunoglobulin deficiencies |
| Patient has human immunodeficiency virus (HIV) and: | Candidiasis of bronchi, trachea, esophagus, or lungs |
|  | Coccidioidomycosis  Cryptococcosis  Cryptosporidiosis, chronic intestinal  Cytomegalovirus disease (particularly CMV retinitis) |
|  | Encephalopathy, HIV-related |
|  | Herpes simplex: chronic ulcer(s) |
|  | Histoplasmosis |
|  | Invasive cervical cancer |
|  | Isosporiasis, chronic intestinal |
|  | Kaposi's sarcoma |
|  | Lymphoma, multiple forms |
|  | Malignant neoplasm |
|  | Mycobacterium avium comp |
|  | Pneumocystis carinii pneumonia  Pneumonia, recurrent |
|  | Progressive multifocal leukoencephalopathy  Salmonella septicemia, recurrent  Toxoplasmosis of brain |
|  | Tuberculosis |
|  | Wasting syndrome due to HIV |
| Drugs | |
| Patient prescribed any: | Abiraterone Acetate |
|  | Aldesleukin |
|  | Alemtuzumab |
|  | Bendamustine |
|  | Bevacizumab |
|  | Bexarotene |
|  | Bortezomib |
|  | Busulfan |
|  | Capecitabine |
|  | Cisplatin |
|  | Cladribine |
|  | Clofarabine |
|  | Crizotinib |
|  | Cyclophosphamide |
|  | Dasatinib |
|  | Durvalumab |
|  | Erlotinib |
|  | Fingolimod |
|  | Ifosfamide |
|  | Imatinib Mesylate |
|  | Ixabepilone |
|  | Lenalidomide |
|  | Mechlorethamine |
|  | Mercaptopurine |
|  | Methotrexate |
|  | Mitoxantrone |
|  | Mycophenolate |
|  | Nilotinib |
|  | Obinutuzumab |
|  | Ofatumumab |
|  | Omacetaxine Mepesuccinate |
|  | Pazopanib |
|  | Peginterferon Alfa-2a |
|  | Pomalidomide |
|  | Ponatinib |
|  | Prednisone |
|  | Regorafenib |
|  | Rituximab |
|  | Romidepsin |
|  | Sirolimus |
|  | Sorafenib Tosylate |
|  | Tacrolimus |
|  | Temozolomide |
|  | Temsirolimus |
|  | Teriflunomide |
|  | Thalidomide |
|  | Thiotepa |
|  | Tofacitinib Citrate |
|  | |
